# Effect of Smear Layer on the Push-Out Bond Strength of Two Endodontic Biomaterials to Radicular Dentin

**Published:** 2013-12-24

**Authors:** Mehrdad Lotfi, Negin Ghasemi, Saeed Rahimi, Mahmood Bahari, Sepideh Vosoughhosseini, Mohammad Ali Saghiri, Vahid Zand

**Affiliations:** a *Research Center for Pharmaceutical Nanotechnology, Department of Endodontics, Dental School, Tabriz University of Medical Sciences,Tabriz, Iran; *; b *Department of Endodontics, Dental School, Tabriz University of Medical Sciences, Tabriz, Iran; *; c *Department of operative dentistry, Dental School, Tabriz University of Medical Sciences, Tabriz, Iran; *; d *Department of Oral and Maxillofacial Pathology, Dental School, Tabriz University of Medical Sciences, Tabriz, Iran; *; e *Research Associated, Department of Ophthalmology and Visual Sciences, University of Wisconsin School of Medicine and Public health, Madison, WI, USA*

**Keywords:** Calcium-Enriched Mixture, Mineral Trioxide Aggregate, Push-Out Bond Strength, Root Canal Preparation, Smear Layer

## Abstract

**Introduction**: This *in vitro* study was designed to evaluate the effect of smear layer removal on push-out bond strength of white mineral trioxide aggregate (WMTA) and calcium-enriched mixture cement (CEM).** Materials and Methods:** Dentin discs with 3 mm thicknesses were divided into 4 groups (*n*=15): group 1: irrigation of the canal with normal saline and filling with WMTA; group 2: irrigation with sodium hypochlorite (NaOCl) and ethylenediaminetetraacetic acid (EDTA), and then filling with WMTA; group 3: same as group 1 but the lumens were filled with CEM; group 4: same as group 2 but the lumens filled with CEM. The samples were incubated at 37^°^C for 3 days after wrapping in gauze pieces moistened with distilled water. The push-out bond strengths were then measured by the universal testing machine and the failure modes were examined under a stereomicroscope at 40× magnification. Data were analyzed using two-way ANOVA and post-hoc Tukey’s test for bond strength. Statistical significance was set at *P*<0.05.** Results:** The greatest and lowest mean standard deviation for push-out bond strength were observed for groups 4 (3.13±1.46 MPa) and group3 (1.44±0.96 MPa), respectively. The effect of smear layer removal on push-out bond strength of CEM was significant (*P*=0.01), however, it was not significant for WMTA (*P*=0.52). The failure mode for all the groups was of mixed type. **Conclusion:** Under the limitations of this study, smear layer removal is recommended for CEM in order to gain higher push-out strength.

## Introduction

As a promising biomaterial, mineral trioxide aggregate (MTA), has been widely used for perforation repair, root canal treatment of immature teeth and as a root-end filling material during apical surgery [1-3]. Despite numerous favorable properties, MTA has some drawbacks including the prolonged setting time and difficult handling characteristics [3]. Recently, calcium-enriched mixture (CEM) cement, which consists of different calcium compounds, was introduced with clinical applications similar to MTA [4]. Compared to MTA, CEM has shorter setting time and better flow, good handling characteristics, and low film thickness [4, 5]. Considering the clinical applications of MTA and CEM, the bond strength of these biomaterials to dentin is an important factor in achieving their best sealing [6]. On the other hand, under dislodging forces, such as functional loads and the pressure related to application of other materials over the first one, the used material should remain in place [7-9]. 

Push-out bond test aims to assess the bond strength of materials to dentin [8, 10]. Reyes-Carmona *et al*. demonstrated that in presence of phosphate-buffered saline, the formation of an interfacial layer (IL) with tag-like structure (TS) and intratubular mineralization (ITM) was evident with MTA [11, 12]. These findings raised the speculation that if the smear layer is removed from the root canal surface, dentinal tubules are exposed to materials. Consequently, the formation of ITM increases and finally high push-out bond strength is expected. Moreover, studies have shown that CEM releases endogenous hydroxyapatite (HA) [5]; therefore, smear layer removal opens the dentinal tubules and thus can lead to TS formation for CEM. Consequently, TS increases the contact surface between the material and dentinal walls and improves mechanical retention by an interlocking phenomenon. However, for WMTA when distilled water is used this phenomenon does not occur due to the lack of phosphate groups. Therefore, this study was conducted to assess the effect of smear layer on the push-out bond strength of WMTA and CEM when distilled water is used as a dressing material.

**Table 1:** The mean push-out bond strength (standard deviation) in MPa for groups

**CEM**		**MTA**	
**With smear layer**	**Without smear layer**	**With smear layer**	**Without smear layer**
1.44 (0.96)	3.07 (1.51)	2.17 (0.72)	2.82 (1.56)

## Material and Methods

Sixty extracted single-rooted human maxillary anterior teeth, stored in 0.5% chloramine-T solution, were selected for this study. Dentin slices from the mid-root area, measuring 3 mm in thickness, were prepared using water-cooled diamond discs (SP1600 Microtome; Leica, Nußloch, Germany). The lumens of the dentin slices were drilled with #2 to #5 Gates-Glidden burs (Dentsply, Maillefer, Ballaigues, Switzerland) to obtain cavities measuring 1.3 mm in diameter. The samples were divided into 4 groups randomly and then mounted on slabs by using sticky wax to simulate the closed-end model. All the specimens were checked to be free of pathways for the irrigation solution to seep out of the lumen. In groups 1 and 3 irrigation was performed using 2 mL of normal saline and the cavity was dried by paper points without contacting canal walls. In groups 2 and 4, the smear layer removal irrigation regimen included: 2 mL of 5.25% sodium hypochlorite (NaOCl) for 10 min, then 2 mL of normal saline, followed by 17% ethylenediaminetetraacetic acid (EDTA) for 5 min; at the end 2 mL of distilled water was used as the final flush and the canals were dried with paper points. In groups 1, 2 and 3, 4 the filling material was ProRoot MTA (Tooth-colored Formula) (Dentsply, Tulsa Dental, Tulsa, OK) (WMTA) and CEM (Yektazist Dandan, Tehran, Iran), respectively. The materials were mixed according to manufacturer's instructions. After obturation of the lumens, the samples were wrapped in gauze pieces moistened with distilled water and incubated for 3 days at 37^°^C. After the experimental period, the samples underwent the push-out bond test. The push-out bond strength was measured using the universal testing machine (Hounsfield Test Equipment, Model H5K-S, Surrey, England). WMTA and CEM were loaded with a 1-mm diameter cylindrical stainless steel plunger at a crosshead speed of 1 mm/min. The maximum load applied to the materials was recorded in Newton (N) before dislodgment occurred and to express the data in MPa, the recorded value in N was divided by area in mm^2^ calculated by the following formula: *2πr.h*, where *π* is the constant 3.14, *r* is the root canal radius, and *h* is the thickness of the root slice in mm. The slices were then examined under stereomicroscope at 40× magnification to determine the mode of the bond failure. Each sample was placed in 1 of the 3 failure modes: adhesive failure that occurred at the material-dentin interface, cohesive failure that happened within the material, and mixed failure mode. Data were analyzed by using two-way ANOVA and post-hoc Tukey’s test for bond strength. *P*<0.05 was assumed as the significance.

## Results

The mean push-out bond strength±standard deviation in MPa for groups is mentioned in Table 1.

The effect of removing of the smear layer on push-out bond strength of CEM was significant (*P*=0.01). However, it was not significant for WMTA (*P*=0.52). There were no significant differences between groups 1, 3 and groups 2, 4 (*P*>0.05). The failure mode for all the experimental groups was of mixed type.

## Discussion

Endodontic materials should be able to remain in place under dislocating forces, such as functional stresses caused by tooth movement during mastication or operative procedures [7, 8]. Therefore, the push-out strength of the materials that are used for vital pulp therapy, as apical barrier and root-end filling materials is important to achieve successful treatment. There are different methods for measuring the adhesion of a dental material to dentin, including tensile, shear, and push-out bond strength tests. The push-out test has been used in this study as a reliable method [8, 10].

This study showed that the push-out bond strength of WMTA and CEM was similar. WMTA and CEM exhibit almost the same expansion during setting [5]. However, other physical and mechanical properties such as flow, film thickness, and setting time were not the same [4, 5]. Removal of the smear layer significantly increased the push-out bond strength of CEM cement in this study. Sarkar *et al*. suggested that calcium ions released by MTA react with phosphate in phosphate-buffered saline (PBS), yielding a HA interfacial layer in the MTA-dentin interface [13]. In addition, MTA releases calcium hydroxide, which interacts with a phosphate-containing fluid to produce calcium-deficient apatite via an amorphous calcium phosphate phase [3]. In addition to verifying the presence of HA interfacial layer, studies have shown the formation of tag-like structures (TS) extending from the intermediate layer to the dentinal tubules, similar to those reported at the resin‒dentin interface [11, 12, 14]. This interlocking improves the mechanical retention of the material used as a plug in the root canal space [12]. Moreover, studies have shown that CEM releases HA endogenously [15]. Therefore, removal of the smear layer opens the dentinal tubules and probably leads to TS formation. Consequently, TS penetration increases the contact surface between the material and dentinal walls and improves mechanical retention by an interlocking phenomenon. However, this study showed that removing the smear layer did not influence the retention of WMTA. This result may be explained by the fact that WMTA cannot produce HA in the presence of water. Thus, open dentinal tubules were not effectively filled by HA. Therefore, the ability of materials to effectively penetrate into the dentinal tubules is one of numerous criteria influencing the selection of materials for root canal treatment. Therefore, it appears that increasing push-out bond strength was not only related to the formation of the interfacial layer but also to the promotion of an ITM process. Moreover, Weng *et al*. reported that the growth and nucleation of the apatite layer are proportional to the concentration of available ions [16]. In fact, in the presence of phosphate components, more phosphate groups are ready to react with calcium ions to create HA interfacial layer and tags in CEM cement. These tags could improve retentive properties of CEM.

In addition, Reyes-Carmona *et al*. have shown the formation of apatite by the combination of MTA and phosphate-buffered saline (PBS). MTA-PBS system was deposited within collagen fibrils, promoting controlled mineral nucleation on dentin and triggering the formation of an interfacial layer with TS at the cement-dentin interface [11, 12, 17, 18]. They concluded that this biomineralization process could be responsible for the over-time improvement in the marginal seal of MTA apical plugs after immersion in PBS. Interestingly, the association of a PBS intracanal dressing and the PBS that diffused through the apical barrier showed greater ITM, displaying the longest and densest TS and lateral branching at different levels of the apical plug [12, 19]. However, in this study, PBS dressing was not used over the materials and interestingly, there was no significant difference between the two biomaterials. It seems that according to the improvement that happened for CEM cement in the presence of phosphate components, if PBS is applied over MTA, higher push-out bond strength is expected for WMTA compared to CEM. However, this concept needs more studies to be confirmed.

From a clinical viewpoint, the biomineralization process supported by the interaction of biomaterials with dentin in the presence of phosphate groups and removal of the smear layer to open dentinal tubules boosts the resistance to displacement of biomaterials from dentin. This process could be responsible for the superior performance reported for the PBS dressing usage [11, 12, 19]. In addition, Lotfi *et al*. reported that adding disodium hydrogen phosphate to WMTA was beneficial to create a more biocompatible material than WMTA alone [18, 20]. Therefore, adding disodium hydrogen phosphate to MTA may be beneficial to produce HA in dentinal tubules. However, this hypothesis needs more studies for confirmation. It can be assumed that removal of the smear layer is recommended for CEM in order to gain higher push-out strength.

## Conclusion

Under the limitations of this *in* *vitro* study, removal of the smear layer did not significantly affect the retention characteristics of WMTA.
